# Bacteria-cancer interactions: bacteria-based cancer therapy

**DOI:** 10.1038/s12276-019-0297-0

**Published:** 2019-12-11

**Authors:** Mai Thi-Quynh Duong, Yeshan Qin, Sung-Hwan You, Jung-Joon Min

**Affiliations:** 1 Laboratory of In vivo Molecular Imaging, Institute for Molecular Imaging and Theranostics, Chonnam National University Medical School and Hwasun Hospital, Jeonnam, 58128 Republic of Korea; 2 Department of Molecular Medicine (BrainKorea21 Plus), Chonnam National University Graduate School, Gwangju, 61469 Republic of Korea; 3 Department of Nuclear Medicine, Chonnam National University Medical School, Jeonnam, 58128 Republic of Korea

**Keywords:** Cancer, Cancer therapy, Cancer, Cancer therapy

## Abstract

Recent advances in cancer therapeutics, such as targeted therapy and immunotherapy, have raised the hope for cures for many cancer types. However, there are still ongoing challenges to the pursuit of novel therapeutic approaches, including high toxicity to normal tissue and cells, difficulties in treating deep tumor tissue, and the possibility of drug resistance in tumor cells. The use of live tumor-targeting bacteria provides a unique therapeutic option that meets these challenges. Compared with most other therapeutics, tumor-targeting bacteria have versatile capabilities for suppressing cancer. Bacteria preferentially accumulate and proliferate within tumors, where they can initiate antitumor immune responses. Bacteria can be further programmed via simple genetic manipulation or sophisticated synthetic bioengineering to produce and deliver anticancer agents based on clinical needs. Therapeutic approaches using live tumor-targeting bacteria can be applied either as a monotherapy or in combination with other anticancer therapies to achieve better clinical outcomes. In this review, we introduce and summarize the potential benefits and challenges of this anticancer approach. We further discuss how live bacteria interact with tumor microenvironments to induce tumor regression. We also provide examples of different methods for engineering bacteria to improve efficacy and safety. Finally, we introduce past and ongoing clinical trials involving tumor-targeting bacteria.

## Cancer: tumor-targeting bacteria enter the anticancer arsenal

Live tumor-targeting bacteria can selectively induce cancer regression and, with the help of genetic engineering, be made safe and effective vehicles for delivering drugs to tumor cells. In a review article, Jung-Joon Min and colleagues from Chonnam National University Medical School in Hwasun, South Korea, discuss the clinical history of using natural or engineered bacterial strains to suppress cancer growth. Because bacteria such as *Salmonella* and *Listeria* preferentially home in on tumors or their surrounding microenvironments, researchers have harnessed these microbial agents to attack cancer cells without causing collateral damage to normal tissues. Bioengineers have also armed bacteria with stronger tumor-sensing and more targeted drug delivery capabilities, and improved control of off-target toxicities. An increasing number of therapeutic bacterial strains are now entering clinical testing, promising to enhance the efficacy of more conventional anticancer treatments.

## Introduction

The challenges faced by current antitumor therapeutics, such as high toxicity to normal cells, the inability to treat deep tumor tissue, and the possibility of inducing drug resistance in tumor cells, have prompted the development of alternative approaches. Many facultative or obligate anaerobic bacteria, such as *Clostridium*, *Bifidobacterium*, *Listeria*, *Escherichia coli*, and *Salmonella* species, possess inherent tumor-targeting and tumor-killing activities. It has been > 100 years since William B. Coley used streptococcal cells and Coley’s toxin to cure patients with inoperable cancers^1^. Further clinical applications using bacteria for treating cancers were curtailed later mainly owing to the emergence of radiation therapy that came into vogue in medical fields since the 1920s. However, recent progress in the fields of immunology and biotechnology has generated new interest in the mechanism underlying the activity of Coley’s toxin, returning bacteria to the forefront for cancer researchers.

Live tumor-targeting bacteria can selectively colonize tumors or tumor-driven lymph nodes, inhibit tumor growth, and prolong survival after systemic infection in animal tumor models. For example, the most well-known attenuated *Salmonella* *Typhimurium* strain VNP20009 is attenuated by more than 10,000-fold compared with the wild-type strain and has a tumor:liver colonization ratio > 1000:1; furthermore, it exhibits robust inhibitory effects on tumor growth and metastasis in mouse models^2,3^. The use of tumor-targeting bacteria as delivery vectors can overcome penetration limitations and maximize the activities of chemotherapeutic drugs while reducing systemic toxicity to the host. Potential payloads for targeted cancer delivery include cytokines, cytotoxic agents, immunomodulators, prodrug-converting enzymes, and small interfering RNAs (siRNAs). By regulating bacterial gene expression, it is possible to further limit the accumulation of antitumor payloads at tumor sites as well as to control the timing of drug delivery.

In this review, we introduce and summarize the technologies underlying bacteria-based anticancer approaches as well as the potential benefits and challenges of these approaches. We also discuss how live bacteria interact with tumor microenvironments (TMEs) to induce tumor regression via colonization and proliferation. Finally, we introduce past and ongoing clinical trials involving tumor-targeting bacteria.

## Mechanisms by which bacteria target and suppress tumors

### Tumor targeting, penetration, and proliferation

The fundamental advantage of bacteria-based cancer therapy is the capability to specifically target tumors via unique mechanisms. For example, using light-emitting attenuated *S*. *Typhimurium* strains defective in ppGpp synthesis (∆ppGpp *S*. *Typhimurium*) and *E. coli* K-12 (MG1655), our group clearly demonstrated that bacteria accumulated exclusively in tumors after intravenous administration in various types of tumor-bearing mice^4–7^. Currently, it is thought that bacteria escape from the blood circulation into tumor tissue via both passive and active mechanisms. Bacteria may initially enter the tumor via passive entrapment in the chaotic tumor vasculature and then flow into the tumor owing to inflammation caused by a sudden increase in the amount of tumor necrosis factor-α (TNF-α) in the tumor vessels^8^. In the TME, the active mechanism likely involves chemotaxis toward molecules produced by dying tumor tissue and the low oxygen concentration in hypoxic tumors, the latter of which might be attractive to obligate anaerobes (e.g., *Clostridium* and *Bifidobacterium*^9,10^) and facultative anaerobes^11,12^. In fact, the active and passive mechanisms are not strain dependent or mutually exclusive, as bacteria might use both pathways to target tumors specifically. The tumor-targeting mechanism of *Listeria* spp. highlights the involvement of the host immune system. *Listeria* cells directly infect not only antigen-presenting cells, such as dendritic cells (DCs) or macrophages but also myeloid-derived suppressor cells (MDSCs), which can then deliver bacteria to TMEs. Through this unique mechanism, *Listeria* cells residing in MDSCs are protected from immune clearance, while *Listeria* cells in healthy tissue milieus are rapidly eliminated^13,14^.

Motility is a critical feature that enables bacteria to penetrate deeper into tumor tissue. Unlike the passive distribution and limited penetration intrinsic to chemotherapeutic drugs, bacteria are complex living organisms that can acquire energy from their surrounding environment; thus, their transport capacity is entropically unlimited. Theoretically, following systemic administration, bacteria can use their self-propulsion abilities to actively swim away from the vasculature to disperse themselves throughout tumor tissue. Forbes et al. observed that *Salmonella* cells started to accumulate in tumors as colonies and spread throughout the entire tumor tissue region within 3 days after injection^15^. Intratumoral *Salmonella* cells show three distinct colonization patterns in tumors: large proliferating colonies formed only near blood vessels and small colonies present both near (inactive) to and far (penetrating) from vessels^16^. Dynamic comparisons of bacterial distribution using in vitro models revealed that motility is critical for effective bacterial dispersion in tumor tissue^17^.

In addition to motility, the host immune response seems to affect the bacterial distribution in tumor tissue. According to a study by Strizker et al.^18^, enterobacterial (e.g., *E. coli* and *S*. *Typhimurium*) tumor colonization is likely influenced by both bacterial metabolism and the host TME, as macrophage depletion resulted in elevated bacterial tumor colonization, while bacterial strains defective for aromatic amino acid biosynthesis showed increased tumor specificity. In regard to the bacteria–host interaction, we previously used microscopy to demonstrate time-dependent changes in the intratumoral distribution of ∆ppGpp *S*. *Typhimurium* cells. The *Salmonella* cells initially spread widely within the tumors; however, as immune cells infiltrated, the *Salmonella* and immune cells interacted with one another, and the bacteria were ultimately surrounded by a neutrophil barrier^7^. Furthermore, as reported by other groups, neutrophil depletion increased the number of intratumoral bacteria and supported bacterial spreading throughout the tumor tissue^8,19^.

After successfully targeting and penetration of tumors, live bacteria can proliferate robustly. In one study using tumor-bearing mice, the number of ∆ppGpp *S*. *Typhimurium* cells reached greater than 1 × 10^10^ CFU/g of tumor tissue 3 days after intravenous administration, with a tumor:normal organ bacterial ratio exceeding 10,000:1^20^; in addition, these bacteria remained countable even after 10 days^21^. The therapeutic *S*. *Typhimurium* strain VNP20009 propagates preferentially in tumors at a ratio of greater than 1000:1 compared with that in normal tissue^2^. Another study reported that *S*. *Typhimurium* strain A1-R selectively proliferates in tumors with a tumor:liver bacterial ratio as high as 2000–10,000:1, while the bacteria were completely cleared from the healthy tissue after 15 days^22^. Although additional studies are needed to clarify why it is beneficial for bacteria to target and grow in tumors, it is undeniable that the ability of therapeutic bacteria to target, penetrate, and proliferate in tumors is a promising advantage that overcomes some of the current limitations of conventional therapies.

### Tumor suppression and microenvironmental changes

**Fig. 1:**
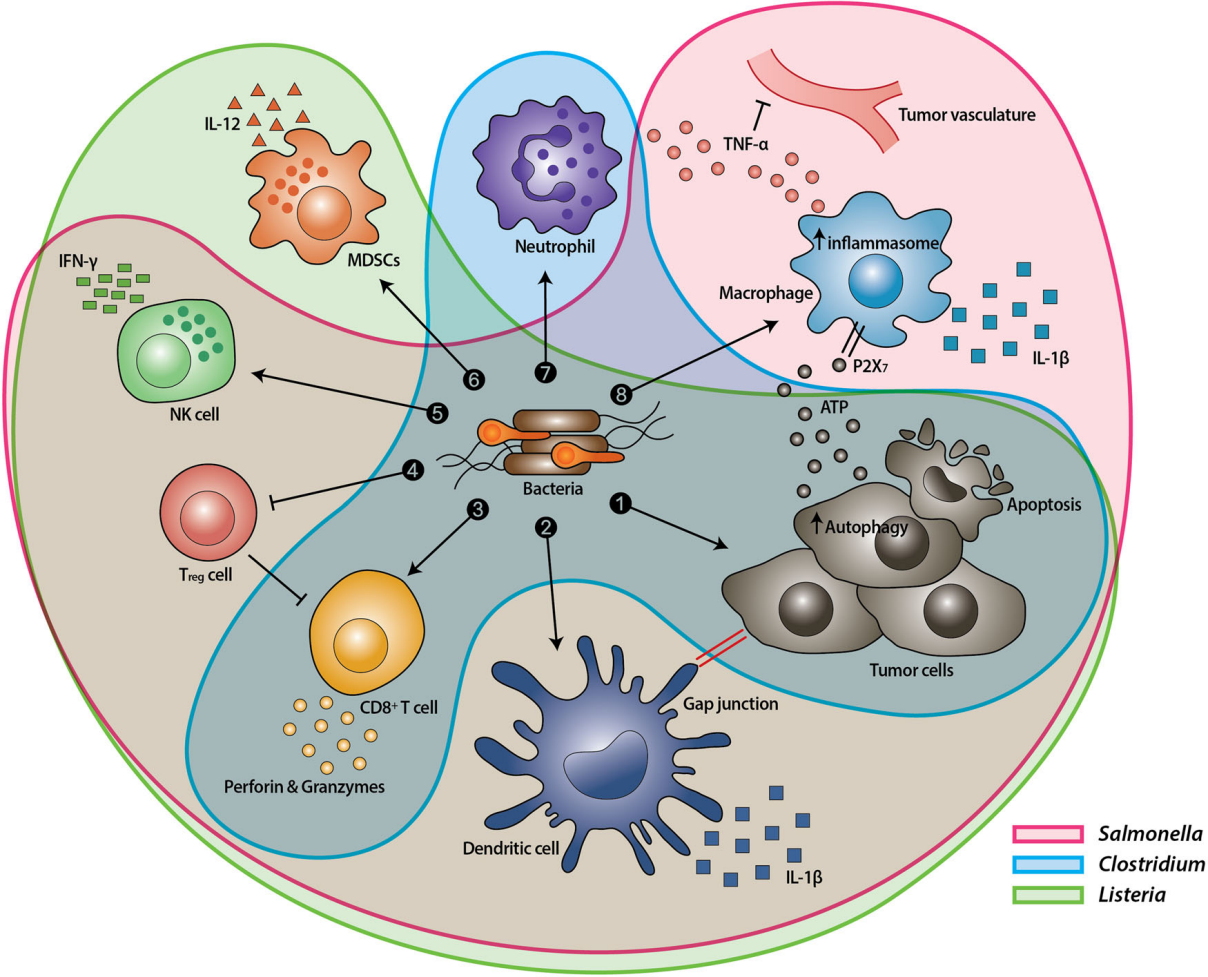
Mechanisms by which bacteria target tumors. After systemic administration, bacteria localize to the tumor microenvironment. The interactions between bacteria, cancer cells, and the surrounding microenvironment cause various alterations in tumor-infiltrating immune cells, cytokines, and chemokines, which further facilitate tumor regression. ① Bacterial toxins from *S*. *Typhimurium*, *Listeria*, and *Clostridium* can kill tumor cells directly by inducing apoptosis or autophagy^15,24,27–30,41^. Toxins delivered via *Salmonella* can upregulate Connexin 43 (Cx43), leading to bacteria-induced gap junctions between the tumor and dendritic cells (DCs), which allow cross-presentation of tumor antigens to the DCs^31–33^. ② Upon exposure to tumor antigens and interaction with bacterial components, DCs secrete robust amounts of the proinflammatory cytokine IL-1β, which subsequently activates CD8^+^ T cells^21,33^. ③ The antitumor response of the activated CD8^+^ T cells is further enhanced by bacterial flagellin (a protein subunit of the bacterial flagellum) via TLR5 activation^39^. The perforin and granzyme proteins secreted by activated CD8^+^ T cells efficiently kill tumor cells in primary and metastatic tumors^33,41^. ④ Flagellin and TLR5 signaling also decreases the abundance of CD4^+^ CD25^+^ regulatory T (T_reg_) cells, which subsequently improves the antitumor response of the activated CD8^+^ T cells^39^. ⑤ *S*. *Typhimurium* flagellin stimulates NK cells to produce interferon-γ (IFN-γ), an important cytokine for both innate and adaptive immunity^31^. ⑥ *Listeria-*infected MDSCs shift into an immune-stimulating phenotype characterized by increased IL-12 production, which further enhances the CD8^+^ T and NK cell responses^14^. ⑦ Both *S*. *Typhimurium* and *Clostridium* infection can stimulate significant neutrophil accumulation^8,20,21,44^. Elevated secretion of TNF-α^8,21^ and TNF-related apoptosis-inducing ligand (TRAIL)^45^ by neutrophils enhances the immune response and kills tumor cells by inducing apoptosis. ⑧ The macrophage inflammasome is activated through contact with bacterial components (LPS and flagellin) and *Salmonella-*damaged cancer cells, leading to elevated secretion of IL-1β and TNF-α into the tumor microenvironment^21,34^. NK cell: natural killer cell. T_reg_ cell: regulatory T cell. MDSCs: myeloid-derived suppressor cells. P2X_7_ receptor: purinoceptor 7-extracellular ATP receptor. LPS: lipopolysaccharide

Bacterial overgrowth in tumors induces tumor regression via several different mechanisms (Fig. 1). Different bacterial strains display distinct mechanisms of tumor suppression in TMEs^23–26^. *Salmonella* spp. kill tumor cells directly by inducing apoptosis and/or autophagy via a variety of mechanisms, including toxin production or deprivation of nutrients from tumor cells^15,27–30^. Furthermore, *Salmonella* infection can induce upregulation of the ubiquitous protein Connexin 43 (Cx43) in tumor cells, promoting gap junction formation between tumor cells and dendritic cells (DCs). These functional connections allow cross-presentation of tumor antigens to DCs, leading to reduced expression of the immunosuppressive enzyme indoleamine 2,3-dioxygenase (IDO) in T cells and a consequential and specific increase in CD8^+^ T-cell activation^31–33^. From the perspective of the host–pathogen interaction, the bacterial components, including lipopolysaccharides (LPS) and flagellin, as well as dynamic bacterial proliferation in tumor masses induce significant migration of innate immune cells, such as macrophages, DCs, and neutrophils, to colonized tumors^8,21^. Subsequently, inflammasome activation leads to robust interleukin-1β (IL-1β) production by macrophages and DCs via two different mechanisms, direct activation by an interaction between *Salmonella* LPS and toll-like receptor 4 (TLR4) and indirect activation owing to the presence of tumor cells that were damaged by *Salmonella*^34^. LPS could be involved in the resulting elevation in TNF-α secretion via interactions with CD14 (a coreceptor of LPS), TLR4, and myeloid differentiation primary response 88^35–37^. Furthermore, flagellin and TLR5 signaling suppress tumor cell proliferation directly^38^ and decrease the number of CD4^+^ CD25^+^ regulatory T cells^39^. Intracellular *Salmonella* flagellin is also involved in NLRC4 inflammasome-driven secretion of IL-1β and IL-18, which serve as activators of IFN-γ-producing cytotoxic T cells and natural killer (NK) cells^40^. Based on accumulating evidence, it is thought that *Salmonella* spp. play a central role in orchestrating complex immune cell alterations during the host antitumor response.

Considering their intrinsic pathogenic properties, *Listeria* spp. can kill tumor cells directly via activation of nicotinamide adenine dinucleotide phosphate oxidase and increased intracellular calcium levels, both of which lead to high reactive oxygen species (ROS) levels^41^. More recently, another study demonstrated a dual mode of action for *Listeria* spp.; *Listeria* cells can enter tumor cells directly or indirectly affect tumors via immunosuppressive effects on MDSCs. *Listeria-*infected tumor cells are then targeted by activated immune cells, whereas an immune-stimulating phenotype is simultaneously induced in a subpopulation of *Listeria-*carrying MDSCs via increased IL-12 production that then supports enhanced T and NK cell responses^14^. Both studies showed that CD8^+^ T cells can efficiently kill tumor cells in primary and metastatic tumors.

During infection by *Clostridium* spp., a variety of secreted bacterial toxins (such as hemolysins and phospholipases) can perturb cellular membrane structure or interfere with intracellular functions^24,42,43^. Like *Salmonella* and *Listeria* spp. infections, clostridial infection can also recruit granulocytes and cytotoxic lymphocytes to TMEs, and such recruitment leads to significant increases in the levels of various cytokines and chemokines at the infected sites that can then promote tumor elimination^44,45^.

In summary, it is speculated that in addition to its intrinsic antitumor effects, bacterial infection makes its most critical contribution to tumor regression by activating a complex immune cell population in TMEs. Although the primary mechanism varies, it is clear that bacteria likely offer a unique immunotherapy strategy that can be potentiated through sophisticated genetic engineering of bacterial strains.

## Engineering of bacteria

### Virulence attenuation

Engineering bacteria to minimize their virulence against the host immune system is also essential^46,47^. It should be noted that some bacterial virulence factors may be responsible for their intrinsic antitumor activity. Therefore, attenuation must be achieved without ablation of their antitumor activity. For example, in human pathogens, fatally toxic strains have been converted into largely safe strains via deletion of major virulence genes^2,10,21^. VNP20009, an attenuated *S*. *Typhimurium* strain, was generated via deletion of the *msbB* and *purI* genes; this strain has been extensively studied in tumor-bearing mice and shows promising tumor-targeting specificity and tumor inhibitory effects^28,48–50^. Deletion of *msbB* in the *Salmonella* genus results in myristoylation of the lipid A component of LPS, which results in a significant reduction in LPS-driven induction of TNF and can reduce the risk of septic shock^51^. VNP20009 was subsequently tested in phase I trials in human cancer patients^51–54^. Disappointingly, VNP20009 lacked tumor specificity and had no clear value in tumor treatment in the patients^52,54,55^. This failure might be attributed to the penta-acylated lipid A produced by VNP20009, which is a TLR4 antagonist^56^. To modify the LPS structure to sustain antitumor activity, mutant *Salmonella* strains were generated that produce a homologous hexa-acylated lipid A, which has a high affinity for TLR4, via deletion of the *pagP*, *pagL*, and *lpxR* genes^47,57,58^. These mutations did not affect virulence in the *msbB* mutant background^59^. Mutations in *rfaG* and *rfaD* result in the production of truncated LPS, which results in attenuated toxicity and tumor specificity; however, the antitumor effects of these bacteria were also decreased. Chromosomal integration of the LPS biosynthetic genes in the *araBAD* locus overcame these limitations, and the mutant strain showed attenuated virulence and therapeutic effects^60^.

Another nontoxic *Salmonella* strain was engineered by downregulating the expression of endotoxin-associated genes or by inhibiting their functional activity. *Salmonella relA-* and *spoT-*mutant strains defective in the synthesis of ppGpp, a signaling molecule involved in toxin gene expression, exhibited negligible toxicity. The LD_50_ value of the ΔppGpp strain was increased up to 10^5^–10^6^-fold compared with those of wild-type strains^61^. The ΔppGpp strain had excellent antitumor activity via its ability to activate the inflammasome (NLRP3, IPAF) and induce the expression of several proinflammatory cytokines (IL-1β, IL-18, and TNF-α)^21^. Deletion of the *phoP* and *phoQ* genes did not affect the antitumor activity of *Salmonella*, while the deletions reduced its virulence in normal tissue^62^. Strains bearing these mutations have been used to produce an excellent vaccine and have recently been used as a delivery system for tumor therapeutics^63–67^. Oral administration of a *S*. *Typhimurium* mutant strain deficient in synthesizing the ZnuABC zinc transport system can induce an effective immune response that protects mice against intestinal infections^68^. Mutations in the genes encoding DNA adenine methylase, adenylate cyclase, and cyclic adenosine monophosphate receptor protein, which are involved in various pathogenic gene regulation pathways, could reduce bacterial toxicity in vivo^69^. Deletion of the *gmd* gene in *Salmonella* inhibited biofilm formation and induced an immune response at an early stage of infection^28,57^. Furthermore, deletion of *htrA*, *STM3120*, and *slyA* significantly reduced bacterial survival in macrophages as well as the anticancer effects of the bacteria^70^. Deletion of genes involved in cell invasion can attenuate *Listeria monocytogenes* cytotoxicity. *Hly* deletion causes defects in phagolysosome release^52,71,72^. *L. monocytogenes* mutant strains lacking *actA* or ActA PEST-like sequences also lack intercellular diffusion ability^73,74^, and mutant strains lacking *inlA* and *inlB* are invasion defective^75,76^.

Introduction of specific nutrient-dependent mutations in bacteria is an additional approach to improving tumor-specific proliferation with virulence attenuation. The A1-R *Salmonella* strain, which is auxotrophic for leucine and arginine, preferentially colonizes tumors, exhibits antitumor effects and increases the susceptibility of tumors to chemotherapy^22,77–79^. A *L. monocytogenes* strain was engineered to be auxotrophic for the cell wall component d-alanine via inactivation of the *dal*/*dat* locus. This mutant strain was highly attenuated and could induce cytotoxic T lymphocytes^22,80^. The *Salmonella* strains SL3261 and SL7207, which carry an *aroA* deletion, and BRD509, which is an *aroA*/*aroD* double mutant strain that is auxotrophic for aromatic amino acids, are highly attenuated and do not disperse freely in the host^29,57,81–85^. Another *S*. *Typhimurium* strain, YB1, which was derived from SL7207 by placing the essential gene *asd* under the control of a hypoxia-induced promoter, could not survive in normal tissue without an exogenous supply of diaminopimelic acid, although it could still colonize hypoxic tumors; thus, this strain causes reduced damage to normal tissue while retaining its tumor-targeting ability^21,47,86,87^. Furthermore, deletion of the *purI* and *purD* genes resulted in the need for exogenous adenine, which increased the ability of the bacteria to efficiently proliferate in purine-rich regions, such as tumor tissue^2,88^. The attenuated bacterial strains used for cancer therapy are listed in Table 1.

**Table 1:** Attenuated bacterial strains used for cancer therapy

Bacteria	Strains	Mutated/modified genes	Phenotype description	References
*Salmonella* *Typhimurium*	A1-R	*∆leu/∆arg*	Auxotrophic strain defective in leucine and arginine synthesis	^22^
	VNP20009	*∆msbB/∆purI*	Lipid A structure modification, reduced ability to induce TNF-α production; deficiency in adenine synthesis	^2^
	SHJ2037	*∆relA/∆spoT*	Unable to produce ppGpp (a global regulator involved in bacterial adaptation to extreme environments); reduction in bacterial invasion	^6,20,21^
	SL3261	*aro-*	Defective in aromatic amino-acid biosynthesis	^83^
	SL7207			^84^
	BRD509			^85^
	YB1			^105^
	LH430; VNP (*Pho/Q-*)	*∆phoP/∆phoQ*	Reduced bacterial survival in macrophages	^60,163^
	MvP728	*∆purD/∆htrA*	Defective in purine biosynthesis and heat-shock protein production in response to stress stimuli	^88^
	YB1; ST8	*∆asd*	Defective in diaminopimelic acid (DAP) synthesis, leading to bacterial lysis during growth without an exogenous DAP supply	^105,164^
	c4550	*∆cya/∆crp*	Disabled production of cAMP (cyclic adenosine monophosphate) synthetase and cAMP receptor protein	^165^
	SF200; S364	*∆pagP/∆pagL/∆lpxR*	Homogenous hexa-acylated lipid A, triggers immune stimulation in the host	^57,58^
	RE88	*∆dam*	Defective in DNA adenine methylase production	^69^
	SB824	*∆sptP*	Defective in pathogenicity island 1 (SPI-1)	^166^
	ST8	*∆gmd*	Limited ability to spread beyond the anaerobic regions of tumors	^164^
	SF200	*rfa-*	Highly truncated LPS and attenuated bacterial virulence	^167^
	MPO378	*∆purD/∆upp*	Defective in purine biosynthesis and uracil phosphoribosyl transferase	^168^
*Listeria monocytogenes*	DP-L4027	*∆LLO (hly)*	Defective phagolysosome release	^71^
	DP-L4029	*∆actA*	Defective surface-bound ActA polypeptide, constitutive LLO activity at physiologic pH	^73^
	DP-L4017	LLO L461T, LLOD26	Cytotoxic, defective cell-to-cell spreading	^52^
	DP-L4042	*∆PEST*	Cytotoxic, defective cell-to-cell spreading	^74^
	DP-L4097	LLO S44A	Cytotoxic, defective cell-to-cell spreading	^72^
	DP-L4364	*∆lplA*	Unable to produce lipoate protein ligase, limited ability to proliferate intracellularly	^169^
	DP-L4405	*∆inlA*	Impaired InlA-mediated infection	^75^
	DP-L4406	*∆nlB*	Impaired InlB-mediated infection	
	CS-L0001	*∆actA/∆inlB*	No host actin nucleation, defective cell-to-cell spreading	^76^
	CS-L0002	*∆actA/∆lplA*		
	CS-L0003	L461T/*∆lplA*	Unable to produce lipoate protein ligase, limited ability to proliferate intracellularly; abortive infection: defective cell-to-cell infection	
	DP-L4038	*∆actA*/L461T LLO	Defective surface-bound ActA polypeptide, constitutive LLO activity at physiologic pH	^52^
	DP-L4384	S44A/L461T	Defective cell-to-cell spreading	

### Enhancement of tumor targeting

The engineering approaches used to improve bacterial tumor targeting can also increase both safety and antitumor efficacy. In one approach to achieve these outcomes, the ppGpp-deficient strain SHJ2037 was genetically engineered to display tumor-specific ligands on the cell surface. An Arg-Gly-Asp peptide that binds to α_v_β_3_ integrin was fused to outer membrane protein A to drive its expression on the bacterial surface^89^. The resulting strain showed enhanced tumor specificity and markedly increased antitumor activity in MDA-MB-231 breast cancer cells and MDA-MB-435 melanoma xenografts overexpressing α_v_β_3_ integrin. Bacteria were also engineered to target tumor-associated antigens, such as carcinoembryonic antigen or the lymphoma-associated antigen CD20. These strains had effective anticancer effects and reduced nonspecific bacterial accumulation in the liver and spleen^90,91^. By exploiting biotin-streptavidin binding, an *L. monocytogenes* strain was constructed in which the cells were coated with plasmid-loaded nanoparticles that expressed a bioluminescence gene. This strain, called a microrobot, delivered functional nucleic acid molecules to solid tumors and could be traced via bioluminescence imaging^60,92,93^. An intriguing alternative that can increase tumor selectivity is to display synthetic adhesins (SAs) on the surface of *E. coli*. SAs have a modular structure consisting of a stable β-domain required for outer membrane anchoring and surface-exposed immunoglobulin domains with high affinity and specificity that can be selected from large libraries^94^. Probiotic strains displayed improved tumor specificity by increasing the injection capacity of the bacteria without attenuating their intrinsic properties^95–99^. Probiotic *E. coli* Symbioflor-2 cells were very rapidly removed from the spleen and liver and survived only in a tumor, indicating efficient tumor targeting. Mice infected with a probiotic *Salmonella* strain tolerated a large bacterial load without any pathological symptoms; however, improvements are needed in the payload delivery system owing to the strain’s inferior therapeutic efficacy, despite its excellent safety in vivo^57^.

### Drug expression strategies

Most payloads delivered by tumor-targeting bacteria are toxic to both tumor cells and normal cells; thus, strict control of their production is preferred over constitutive expression. Precise triggering of payload expression can maximize its therapeutic effects while minimizing systemic toxicity. A controllable gene expression system can theoretically be constructed by inserting a specific promoter sequence upstream of a drug-encoding gene, thereby conferring transcriptional control via external signals. Such a system makes it possible to manage the timing and location of drug production in vivo. The strategies for this type of gene regulation, or triggering, belong to mostly following three categories: internal triggering, self triggering (quorum sensing-QS), and external triggering^100^.

Unlike normal tissue, TMEs have special properties, including hypoxia, acidosis, and necrosis, which bacteria can sense and utilize to improve tumor specificity^101^. For example, hypoxia-inducible promoters such as those of HIP-1 and *pepT* are activated by fumarate and nitrate reduction in the hypoxic environment within tumor tissue^102–104^. This hypoxia-inducible expression system was designed to function under only anaerobic conditions for expression of essential genes such as *asd*^105^. Flentie et al. identified five promoter sequences specifically activated by the acidic microenvironments associated with cancer cells in vitro and with tumors in vivo. Acidosis-specific promoters were identified by using a custom-designed promoterless transposon reporter encoding bacterial luciferase to screen a library of 7400-independent *Salmonella* transposon insertion mutants in coculture with melanoma or colon carcinoma cells. An attenuated *Salmonella* strain expressing Shiga toxin under the control of a promoter induced by low pH showed strong tumor selectivity and antitumor activity^106^. A glucose sensor was engineered in *E. coli* via synthetic fusion of the Trg chemoreceptor with the EnvZ osmosensor. In this construct, Trg contributes the periplasmic and transmembrane domains as well as a short cytoplasmic segment, and EnvZ contributes the cytoplasmic kinase/phosphatase domain. The engineered bacteria sensed the glucose levels in tumor cell masses and responded to the tumor’s metabolic activity, possibly leading to its therapeutic effect. These features are likely conserved in other members of this sensor family^107^.

Bacteria can colonize and proliferate in TMEs at a tumor-to-normal tissue ratio exceeding 10,000; thus, QS can be used as a gene expression switch^108,109^. One useful QS system is regulated by an autoinducer, the synthetic LuxI protein, and the transcriptional regulatory protein LuxR. The acylhomoserine lactone (AHL) produced by LuxI, which depends on bacterial density, activates LuxR and promotes transcription of its target genes. AHL concentration-dependent QS systems have been used successfully to highly express heterologous proteins in bacteria-colonizing tumors^90,109–111^. The QS approach has been used to introduce a variety of gene circuits. For example, introduction of a synchronized lysis circuit into bacteria improved anticancer efficacy by allowing drug release via periodic introduction of the autoinducer (positive feedback) and the resulting activation of a bacteriophage lysis gene (negative feedback)^108^.

In addition to internal and self triggering, the expression of gene circuits can also be controlled by external inducers, including chemicals such as l-arabinose, salicylic acid (ASA), and tetracycline. Transcription from the P_BAD_ promoter can be controlled via an interaction between the AraC repressor and l-arabinose^5,112^. In an attenuated *Salmonella* strain, pBAD-driven expression of therapeutic payloads from a plasmid could be regulated via intravenous or intraperitoneal l-arabinose administration^20,21,113^. A *Salmonella* strain with a mutation in the Ara operon, which results in impaired l-arabinose metabolism, exhibits strong activation of the P_BAD_ promoter^114^. In the ASA expression system, gene regulation is controlled by the XylS2-dependent Pm promoter^115–117^. An attenuated *Salmonella* strain harboring an ASA expression system on a plasmid or on the chromosome allowed efficient regulation of genes encoding prodrug-converting enzymes (see below) and led to a marked reduction in tumor growth^115^. The pTet expression system is simultaneously regulated by the P_tetA_ and P_tetR_ bidirectional promoters, which are induced by tetracycline or doxycycline^118^. In a preclinical study, a reporter gene and a therapeutic gene were inserted under these bidirectional promoters to visualize the targeting process and deliver therapeutic drugs, respectively^7^. This chemically inducible system is regulated in a dose- and time-dependent manner^119^; therefore, inaccuracies in the timing of inducer administration or the dose of the inducer can lead to nonspecific or suboptimal expression of the target molecules in TMEs. An alternative inducible system uses the radiation-inducible *recA* promoter^48^. Radiation causes DNA damage that activates transcription of the genes under the control of the *recA* promoter. This method combines the therapeutic effects of radiation therapy with radiation-dependent induction of anticancer gene expression^120^. Induction of TNF-related apoptosis-inducing ligand (TRAIL) expression via a 2 Gy dose of γ-irradiation 48 hours after administration of the engineered *Salmonella* strain significantly delayed the growth of 4T1 breast cancer cells^48^. Radiation is advantageous because it can penetrate the tumor tissue and be used for localized treatment. However, radiation can also cause toxicity by inducing DNA damage in the healthy cells around the tumor and likely causes fatal mutations in the therapeutic bacteria that could attenuate their therapeutic effect^101^.

### Drug delivery

Despite studies on the antitumor effects of bacteria, bacteria alone are often insufficient to suppress tumors completely. To enhance the positive outcomes of bacterial cancer therapy, the usefulness of eukaryotic and prokaryotic expression systems for the delivery of therapeutic payloads, including cytotoxic agents, prodrug-converting enzymes, immune regulators, tumor stroma-targeting molecules, and siRNAs, has been explored (listed in Table 2). The prokaryotic expression systems discussed above are the most commonly used approach, and these systems depend on transforming bacteria with prokaryotic plasmids encoding target genes^70,121,122^; by contrast, eukaryotic expression systems involve transduction of host cells, such as immune cells or tumor cells, with eukaryotic plasmids encoding the cDNAs of the target genes^123^.

**Table 2:** Payloads for bacteria-mediated drug delivery

Antitumor agents	References
Strategies	
Hypoxia-inducible promoter	^102–104^
Acidosis-specific promoter	^106^
Glucose-dependent hybrid receptor Trz1	^107,170^
Quorum sensing	^90,108–111^
l-arabinose-inducible pBAD promoter	^5,20,21,95,112,114^
Salicylic acid-inducible Pm promoter	^115–117^
Tetracycline-inducible Tet promoter	^7,118^
Radiation-inducible RecA promoter	^48,120^
Cytotoxic agents	
ClyA	^5,7^
Apoptin	^65^
TNF-α	^120,127^
TRAIL	^48,78,126,171^
FasL	^49^
Invasin	^128^
Azurin	^29^
Prodrug-converting enzymes	
CD	^54^
HSV1-TK/GCV	^131^
β-glucuronidase	^129^
Carboxypeptidase G2	^130^
Immunomodulator	
IL-18	^49^
IL-2	^23,29^
FlaB	^20,141^
PSA	^84,139,144^
HER-2/neu	^143^
NY-ESO-1	^83^
Survivin	^88^
Mage-b	^145^
Tumor stroma	
Endostatin	^148,149^
VEGFR2	^121,145^
Endoglin	^150,151^
siRNA	
Stat3	^63,122,149,152^
IDO	^153^
Survivin	^155^
Sox2	^30^
PLK1	^57^

*ClyA* cytolysin A, *TNF-α* tumor necrosis factor-α, *TRAIL* TNF-related apoptosis-inducing ligand, *FasL* Fas ligand, *CD* cytosine deaminase, *HSV1-TK/GCV* herpes simplex virus type I thymidine kinase/ganciclovir, *IL-18* interleukin-18, *IL-2* interleukin-2, *FlaB* flagellin, *PSA* prostate-specific antigen, *VEGFR2* vascular endothelial growth factor receptor, *Stat3* signal transducer and activator of transcription 3, *IDO* immunosuppressor indoleamine 2,3-dioxygenase, *PLK1* cell cycle-associated polo-like kinase 1

#### Cytotoxic agents

Cytotoxic agents carried by tumor-targeting bacteria can have intrinsic antitumor activity. Combined with the use of inducible promoters, the expression of cytotoxic agents can be tightly controlled to reduce their toxic effects on normal tissue. Cytolysin A (ClyA), a 34 kDa pore-forming hemolytic protein produced by *E. coli*, *S*. *Typhimurium*, and *Paratyphi A*, can be transported to the bacterial surface and secreted without posttranslational modification. Several bacterial strains, such as *E. coli* and attenuated *S*. *Typhimurium*, have been engineered to express ClyA from a constitutive promoter^5^ or from inducible promoters activated by arabinose^5^ or doxycycline^7^, and these strains have shown excellent tumor-inhibiting effects.

Induction of apoptosis in tumor cells is a promising cancer therapy strategy. Apoptin, a chicken anemia virus-derived protein, selectively induces apoptosis in a large number of human cancer cell types through a p53-independent, Bcl-2-insensitive pathway, with no effects on normal tissue^124^. By transforming an apoptin-encoding eukaryotic expression plasmid (pCDNA3.1) into an attenuated *S*. *Typhimurium* strain, Wen et al. observed significant tumor regression with minimal systemic toxicity in human laryngeal cancer-bearing mice^65^. Other cytotoxic agents that can be similarly used to induce apoptosis include three members of the TNF-α family (TNF-α, TRAIL, and the Fas ligand); however, owing to their short half-life and hepatotoxicity, the usefulness of these cytotoxic ligands is limited by their insufficient tumor exposure and detrimental effects on liver function^125^. To improve the bioavailability and sustainability of these proteins, bacteria have been used to deliver them directly to the tumor region^48,78,126,127^. Forbes et al. engineered a nonpathogenic *S*. *Typhimurium* strain expressing murine TRAIL under the control of the radiation-inducible *recA* promoter. After irradiation, TRAIL was secreted, whereupon it delayed mammary tumor growth significantly and reduced the risk of death^48^.

Invasin, a *Yersinia* surface protein, can bind to β1 integrin selectively to trigger bacterial entry into host cells. Using a nonpathogenic, recombinant invasive *E. coli* strain coexpressing invasin and the model antigen ovalbumin as well as LLO, Critchley-Thorne et al. showed that the engineered strain could invade β1 integrin-expressing cells and deliver proteins to tumors to produce therapeutic effects in mice^128^. Azurin, a low-molecular-weight redox protein, can be internalized efficiently to initiate cancer cell apoptosis by raising the intracellular p53 and Bax levels to induce the release of mitochondrial cytochrome c into the cytosol. The effectiveness of *E. coli-*based azurin delivery in suppressing the growth of B16 mouse melanoma and 4T1 mouse breast cancer was demonstrated by Nissle in 1917; furthermore, this approach also prevented pulmonary metastasis and stimulated inflammatory responses^29^.

#### Prodrug-converting enzymes

The expression of prodrug-converting enzymes can convert prodrugs into cytotoxic agents specifically in the tumor region. The usefulness of this strategy to improve cancer treatment efficacy and reduce the side effects associated with systemic administration has been explored. Several prodrug-converting enzymes have been delivered by bacteria^96,129–131^. Cytosine deaminase (CD) converts nontoxic 5-fluorocytosine (5-FC) into the chemotherapeutic agent 5-fluorouracil (5-FU). 5-FU is highly toxic because it is further metabolized into a product that interferes with DNA and RNA synthesis^132–135^. Upon coadministration of an attenuated *S*. *Typhimurium* (VNP20009) strain expressing *E. coli* CD and 5-FC into patients, conversion of 5-FC to 5-FU was observed, indicating bacterial production of functional CD in the tumor^54^.

The herpes simplex virus type I thymidine kinase/ganciclovir (HSV1-TK/GCV) system is another prodrug-converting enzyme/prodrug combination that has been widely studied for use in tumor therapy. Tumor tissue-specific HSV1-TK expression can convert the nontoxic precursor ganciclovir into ganciclovir-3-phosphate, a toxic substance that kills tumor cells. Liu et al. tested the in vivo efficacy of a *Bifidobacterium infantis* strain expressing HSV1-TK and GCV for prodrug therapy in a rat bladder tumor model. The results showed that this targeted approach could effectively inhibit rat bladder tumor growth by increasing caspase 3 expression and inducing apoptosis^131^. Another prodrug-converting enzyme delivery strain, *E. coli* DH5α expressing β-glucuronidase, which hydrolyzes the glucuronide prodrug 9ACG into the topoisomerase I inhibitor 9-aminocamptothecin (9AC), showed efficient tumor inhibition^129^. The use of attenuated *S*. *Typhimurium* VNP20009 as a vector to deliver carboxypeptidase G2 showed enhanced antitumor efficacy in conjunction with prodrug administration^130^.

#### Immunomodulators

Cytokines can achieve antitumor effects by facilitating the proliferation, activation, and differentiation of immune cells, by inducing apoptosis in tumor cells, and via antiangiogenesis effects on tumor vasculature. Several cytokines, including GM-CSF, IL-12, and IL-18, have entered clinical trials for cancer therapy^136^. Cytokines expressed by tumor-targeting bacteria were delivered specifically to the tumor region, where they augmented the antitumor immune response in the TME^20,29,49,62,137–140^. Intravenous administration of an IL-18-expressing attenuated *S*. *Typhimurium* strain inhibited primary tumor growth in mice, induced massive leukocyte infiltration (mainly granulocytes), and increased NK and CD4^+^ T cell but not CD8^+^ T-cell recruitment. Furthermore, this approach also increased cytokine production in the tumor region, including that of IL-1β, TNF-α, IFN-γ, and GM-CSF^49^. IL-2 is the most widely studied cytokine in the context of bacterial delivery systems. Oral administration of a *S*. *Typhimurium* Ty21a strain expressing IL-2 inhibited hepatocellular carcinoma (HCC) in mouse models^23,29^. Flagellin, which activates the innate immune system via TLR5, has been established as an excellent immunotherapy adjuvant^141^. Our group treated colon cancer-bearing mice with an attenuated ΔppGpp *S*. *Typhimurium* strain expressing heterologous flagellin and showed that it enhanced antitumor immunity via cooperation with the TLR4 and TLR5 signaling pathways. This approach also promotes an M2-to-M1 shift in macrophages and increases nitric oxide levels in tumors^20^.

Engineered bacteria expressing tumor-associated antigens may sensitize TMEs and overcome the self tolerance aroused by regulatory T cells, thereby eliciting effector and memory T-cell responses toward antigen-producing tumor cells^142,143^. A number of prostate cancer-associated antigens have been reported. Bacteria-based vaccines against prostate-specific antigen (PSA) have been tested in several mouse models^139,144^. Endogenous PSA gene delivery using attenuated *S*. *Typhimurium* SL7207-alleviated immune tolerance to murine prostate stem cell antigens and significantly retarded tumor growth^84^. Gene therapy approaches using antigens against HER-2/neu^143^, NY-ESO-1^83^, survivin^88^, and Mage-b^145^ have also shown promising tumor inhibition effects.

Great interest has developed in the field of immune checkpoint blockade (ICB) cancer therapy. Despite the success of ICB therapy in clinical trials, only some patients benefit from this treatment. There are several reasons for the host resistance underlying this effect, of which the immunosuppressive TME is the most important^146,147^. Studies demonstrate that tumor colonization by bacteria can induce proinflammatory reactions involving elevated expression of IL-1β, TNF-α, and IFN-γ, as well as NK and T-cell activation; therefore, a combination of ICB and bacterial therapies may overcome host resistance^131^.

#### Targeting the tumor stroma

Angiogenesis plays important role in tumor growth and metastasis. Targeting tumor neovascularization provides a promising direction for cancer therapy. Endostatin is a 20 kDa C-terminal fragment from type XVIII collagen that can inhibit tumor vessel generation in a dose-dependent manner without obvious side effects or drug resistance^148,149^. Xu et al. cloned endostatin and an siRNA against signal transducer and activator of transcription 3 (Stat3) in an attenuated *S*. *Typhimurium* strain. They then tested the strain’s therapeutic efficacy in orthotropic HCC and showed that it could inhibit tumor proliferation and metastasis, reduce the amount of tumor microvasculature, increase the CD4^+^/CD8^+^ T-cell populations and the expression levels of several inflammatory cytokines (including IFN-γ and TNF-α), and downregulate TGF-β, regulatory T cells, and vascular endothelial growth factor (VEGF) expression^149^. VEGF and its receptor (VEGFR) regulate tumor angiogenesis^121,145^. Oral administration of attenuated *S*. *Typhimurium* SL3261 expressing the extracellular VEGFR2 domain inhibited tumor growth, neovascularization, and pulmonary metastasis. Furthermore, the percentages of CD4^+^ and CD8^+^ T cells in the tumor region also increased significantly^121^. Endoglin (CD105) is a member of the TGF-β receptor family. TGF-β1 and hypoxia can upregulate the endoglin gene promoter, and this promoter is highly active in tumoral endothelial cells. Therefore, endoglin has been considered a target for cancer therapy^150^. Paterson et al. used *Listeria*-based vaccines against CD105, Lm-LLO-CD105A, and Lm-LLO-CD105B to treat breast cancer in a mouse model. The vaccines stimulated a robust antiangiogenesis effect and an antitumor immune response that inhibited primary and metastatic tumors^151^.

#### Gene silencing

siRNAs, a class of 20–25 base pair-long double-stranded RNAs that mediate silencing of specific target genes, have provided a promising approach to cancer therapy. However, the largest barrier to RNA interference therapy is the need for specific delivery of siRNAs to the tumor region. Bacteria-based delivery systems for siRNAs against Stat3^63,122,149,152^, IDO^153,154^, survivin^155^, Sox2^30^, and the cell cycle-associated polo-like kinase 1 (PLK1)^57^ have been tested in mouse tumor models. Oral administration of an attenuated *S. Typhimurium* strain harboring a eukaryotic expression plasmid encoding siRNA-Stat3 enhanced NK cell activity and T-lymphocyte function and elevated the percentage of CD8^+^ T cells, whereas it decreased the number of CD4^+^ CD25^+^ regulatory T cells in the tumor; these effects led to inhibition of tumor growth and prolonged survival of tumor-bearing mice^122^. Silencing host IDO expression using a *S*. *Typhimurium* VNP20009 strain expressing shIDO elicited significant tumor infiltration by ROS-generating polymorphonuclear neutrophils, which promoted intratumoral cell death and substantial control of B16F10 melanomas^153^ and CT26 or MC38 colorectal cancers^156^.

## Clinical Trials

Since Dr. William B. Coley first used the live infectious agent erysipelas (*Streptococcus pyogenes*) for cancer treatment in 1891^157^, several bacterial strains have been studied and selected for testing in human patients (Table 3). Among the bacterial species selected for human studies, *Listeria* vaccine strains (with or without combination agents) have shown very promising outcomes, and some strains are now being tested in phase II and III clinical trials^158^.

**Table 3:** Previous and ongoing clinical trials

Bacterial strain	Phase	Cancer type	*n*	References
*S*. *Typhimurium* VNP20009	I	Metastatic melanoma; metastatic renal cell carcinoma	25	^159^
*S*. *Typhimurium* VNP20009	I	Melanoma	4	^53^
*S*. *Typhimurium* VNP20009 expressing TAPET-CD (cytosine deaminase)	I	Head and neck or esophageal adenocarcinoma	3	^54^
*S*. *Typhimurium* VNP20009	I	Patients with advanced or metastatic solid tumors	Not provided	http://www.clinicaltrials.gov/ct2/show/NCT00004216
*S*. *Typhimurium* VNP20009	I	Unspecified adult solid tumors	Not provided	https://www.clinicaltrials.gov/ct2/show/NCT00006254
*S*. *Typhimurium* VNP20009	I	Neoplasm or neoplasm metastatic tumors	45	http://www.clinicaltrials.gov/ct2/show/NCT00004988
*S*. *Typhimurium* expressing human IL-2	I	Liver cancer	22	https://www.clinicaltrials.gov/ct2/show/NCT01099631
*S. Typhimurium* Ty21a VXM01	I	Pancreatic cancer	26	^172^
*Clostridium novyi*-NT	I	Colorectal cancer	2	https://www.clinicaltrials.gov/ct2/show/NCT00358397
*Clostridium novyi*-NT	I	Solid tumor malignancies	5	https://www.clinicaltrials.gov/ct2/show/NCT01118819
*Clostridium novyi*-NT	I	Solid tumor malignancies	24	https://www.clinicaltrials.gov/ct2/show/NCT01924689
*Clostridium novyi*-NT	Ib	Refractory advanced solid tumors	18- recruiting	https://clinicaltrials.gov/ct2/show/NCT03435952
*Listeria monocytogenes*	II	Metastatic pancreatic tumors	90	^173^
*Listeria monocytogenes*	II	Cervical cancer	109	^174^
*Listeria monocytogenes*	III	Cervical cancer	450- recruiting	https://clinicaltrials.gov/ct2/show/record/NCT02853604

In 1999, the attenuated *S*. *Typhimurium* VNP20009 strain designed by Vion Pharmaceutics, Inc., was the first *Salmonella* strain to enter phase I human clinical trials. The effectiveness of this strain was tested in 24 patients with metastatic melanoma and in one patient with metastatic renal carcinoma. Analysis of increasing doses (1 × 10^6^–1 × 10^9^ CFU/m^2^ delivered via intravenous injection) revealed that the maximum-tolerated dose) was 3.0 × 10^8^ CFU/m^2^. Although increased levels of several proinflammatory cytokines (such as IL-1β, TNF-α, IL-6, and IL-12) and tumor colonization were found in some patients, no objective tumor regression was observed, even in patients with colonized tumors^159^. Another clinical trial with *S. Typhimurium* VNP20009 was performed with four additional metastatic melanoma patients, but there was no objective tumor response, and only minor and transient side effects were observed^53^. To improve its therapeutic efficacy, VNP20009 was modified to express *E. coli* CD, which converts 5-FC to toxic 5-FU. Three patients with head and neck squamous carcinoma and esophageal adenocarcinoma were treated by intratumoral injection of these bacteria (at three increasing doses: 3 × 10^6^, 1 × 10^7^, or 3 × 10^7^ CFU/m^2^) for multiple cycles; 100 mg/kg/day 5-FC was delivered orally three times daily for multiple cycles. Two patients showed tumor colonization for at least 15 days after the initial administration, with a tumor-to-plasma ratio of 3:1; this ratio was <1.0 in the noncolonized patient. No significant adverse responses were observed after six treatment cycles. More recently, there have been four other unpublished and completed phase I clinical trials using *S*. *Typhimurium* (three clinical trials with attenuated VNP20009 and one clinical trial with *S*. *Typhimurium* χ4550 expressing IL-2, as summarized in Table 3). The results of these clinical trials revealed that discrepancies between the outcomes in preclinical animal models and human patients might be owing to differences in tumor structure and growth rates that could alter bacterial penetration, proliferation, and clearance within tumors as well as in the peripheral circulation. Another lesson learned from the clinical trials using *Salmonella* spp. is that TLR4-mediated signaling might be important for tumor colonization and antitumor activity, as a VNP20009 strain lacking lipid A function failed to colonize tumors sufficiently enough to suppress tumor growth (as discussed above).

Based on the tumor colonization and tumor lysis studies initiated by Möse and colleagues in the 1950s^160,161^, the oncolytic *Clostridium butyricum* M-55 strain (also known as *Clostridium sporogenes* ATCC 13732) has entered phase I clinical trials with a large number of patients. Robert et al.^162^ demonstrated promising antitumor responses in both canine and human clinical studies after intratumoral injection of *Clostridium novyi*-NT spores. In this study, the data from lesion biopsies and computed tomography imaging clearly showed extensive tumor destruction owing to gas pockets produced by *C. novyi*-NT. Although tumor colonization and objective tumor responses were observed using both intravenous and intratumoral administration in these clinical trials, the *Clostridium* cells alone failed to eradicate all of the cancer cells, resulting in tumor relapse. Currently, a phase Ib clinical trial using a *C. novyi*-NT strain in combination with an anti-PD1 antibody (pembrolizumab) to treat refractory advanced solid tumor patients is underway (summarized in Table 3).

Although limited, these clinical data have revealed many important obstacles as well as encouraging challenges that must be overcome for successful human application in the future. Perhaps engineering bacteria to specifically target tumors or the use of combinations of bacteria-based approaches and other immunotherapies in conjunction with advanced diagnostic approaches will enable better intratumoral bacterial colonization and enhance the resulting therapeutic outcomes.

## Conclusions and future perspectives

Tumor-targeting bacteria possess unique features, including tumor selectivity and unlimited gene packaging capability, that make them ideal vehicles for delivering therapeutic payloads in a cancer-specific manner. This unlimited gene packaging capability not only allows the expression of large or multiple target genes but also supports engineering signaling networks that can enable bacteria to perform sophisticated tasks in cancer treatment. Despite the great therapeutic potential of engineered tumor-targeting bacteria, successful cancer therapy will likely require combinatorial approaches, as cancer heterogeneity (at both the molecular and histologic levels) makes it very difficult to achieve a cure with single anticancer agents. In addition to chemotherapy and radiotherapy, whose anticancer effects can be synergistic with those of bacteria, intratumoral bacterial infection is attractive as an amendment to other immunotherapeutic approaches. For example, some natural or engineered bacterial strains can induce a tumor-specific T-cell response in TMEs or lymphoid tissue via activation of multiple TLR pathways, induction of bacteria-specific CD4^+^ T cells, and generation of proinflammatory TMEs. This approach may have unique characteristics that enable the bacteria to induce TLR-mediated CD8^+^ and CD4^+^ T-cell infiltration of poorly infiltrated tumors, thereby leading to a synergistic effect with ICB treatment. More-sophisticated engineering of tumor-targeting bacteria may allow stronger tumor sensing, finer tuning of drug production, and improved control of bacterial toxicity and genetic instability. Clinical studies with such “smart” bacteria will hopefully establish this approach as another powerful weapon in the arsenal in our fight against cancer in the near future.
